# Noise trauma and systemic application of the selective glucocorticoid receptor modulator compound A

**DOI:** 10.1186/s12952-016-0053-0

**Published:** 2016-05-11

**Authors:** Lukas D. Landegger, Clemens Honeder, Chengjing Zhu, Hanna Schöpper, Elisabeth Engleder, Franz Gabor, Wolfgang Gstoettner, Christoph Arnoldner

**Affiliations:** Department of Otorhinolaryngology, Medical University of Vienna, Waehringer Guertel 18-20, 1090 Vienna, Austria; Department of Pathobiology, Institute of Anatomy, Histology and Embryology, University of Veterinary Medicine, Veterinaerplatz 1, 1210 Vienna, Austria; Department of Pharmaceutical Technology and Biopharmaceutics, University of Vienna, Althanstrasse 14, 1090 Vienna, Austria

**Keywords:** Selective glucocorticoid receptor modulators, SEGRMs, Compound A, Dexamethasone, Guinea pigs, Otoprotection, Noise-induced hearing loss

## Abstract

**Background:**

Selective glucocorticoid receptor modulators (SEGRMs) comprise a novel class of drugs promising both reduced side effects and similar pharmacological potency relative to glucocorticoids, which presently serve as the only clinical treatment for many otologic disorders. In the first otologic SEGRM experiment in an animal model of noise trauma, we compare the effects of Compound A (a SEGRM) and dexamethasone (potent glucocorticoid).

**Methods:**

Forty adult guinea pigs received experimental treatment once daily for ten days. The animals were divided into four cohorts based on the treatment received: Compound A (1 mg/kg or 3 mg/kg), dexamethasone (1 mg/kg) as gold standard, or water as negative control. After five applications, animals were exposed to broadband noise (8–16 kHz) at 115 dB for three hours. Hearing thresholds were determined by recording auditory brainstem responses to clicks and noise bursts (1–32 kHz) and were assessed a week prior to and immediately after exposure, as well as on days 1, 3, 7, 14, 21, and 28. Cochleae were prepared as whole-mounts or embedded and sectioned for histological analysis.

**Results:**

Relative to the control treatments, Compound A failed to preserve auditory thresholds post-noise exposure with statistical significance. Histological analyses confirm the physiological result.

**Conclusion:**

The present findings suggest that Compound A does not have substantial otoprotective capacities in a noise trauma model.

## Background

Glucocorticoids currently serve as the only clinically available treatment for a variety of otologic disorders. However, they often produce severe side effects including diabetes, short-term blood glucose level dysregulation, osteoporosis, and stunted growth [1–4]. To minimize these risks, it is now common for steroids to be applied locally (e.g., intratympanically) for therapy, but there is still a clinical need for more effective and specific compounds and a better understanding of how glucocorticoids exert their otoprotective effects [5, 6].

Two processes have been identified as particularly significant in glucocorticoid activity: *transrepression* and *transactivation. Transrepression* of proinflammatory transcription factors (e.g., NF-KB) is triggered when cytosolic glucocorticoid receptors (GRs) bind the active agent and the ligand-receptor complex translocates to the nucleus [7]. *Transactivation* summarizes the dimerization of GRs and the subsequent binding to specific DNA sequences (the glucocorticoid response element or GRE), which primarily causes the side effects associated with glucocorticoid use. Selective glucocorticoid receptor modulators (SEGRMs – until recently uniformly referred to as SEGRAs/-agonists) were developed to counter inflammation by interfering with the transcription factor pathway (receptor monomers) without influencing the GRE, thus decreasing the likelihood of adverse events (although this hypothesis has become controversial in recent years) [8].

Extensive in vitro and in vivo research has demonstrated that the first commercially available SEGRM, Compound A (CpdA), favors *transrepression* over *transactivation* [8]. CpdA’s anti-inflammatory effects have been demonstrated in arthritis, asthma, and inflammatory bowel and neuroinflammatory disease models, with several studies showing reduced side-effect profiles [9–16]. However, CpdA’s efficacy has not yet been tested in the ear. On the contrary, many research groups have applied synthetic glucocorticoids (e.g., methylprednisolone or dexamethasone) to the ear in both animal and human models to assess their effects on temporary threshold shifts (TTS: <24 h) and/or permanent threshold shifts (PTS: 2–3 weeks later), albeit with varying degrees of success [17–20].

In the first, to the best of our knowledge, study testing a SEGRM in otology, our group demonstrated that intratympanic CpdA delivery resulted in hearing loss in a guinea pig model, whereas systemic application did not produce threshold shifts, suggesting a stabilizing effect of plasma protein binding [21, 22].

The current trial was designed to determine whether CpdA could serve as a systemic alternative with a potentially more favorable side-effect profile, i.e., an agent triggering fewer of the above-mentioned complications.

## Methods

All animal procedures were approved by the local Institutional Animal Care and Use Committee and the Austrian Federal Ministry for Science and Research (BMWF-66.009/0165-II/3b/2013). Rodent care and handling were in accord with the Federation of European Laboratory Animal Science Associations’ guidelines.

**Fig. 1:**
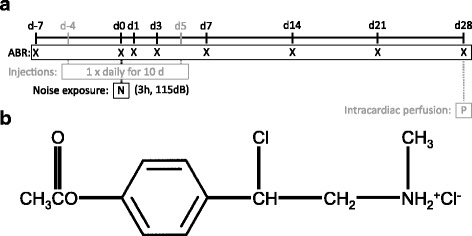
**a** Experimental timeline. Abbreviations: d = day/s, ABR = auditory brainstem reponse, h = hours, dB = decibels. **b** Chemical structure of Compound A

40 adult pigmented guinea pigs were divided into 4 cohorts of 10 animals each, controlling for gender (M = 5; F = 5 in each cohort) and weight (all animals ≥300 g in weight, no statistically significant difference between cohorts). Rodents received an intraperitoneal injection of one of four experimental treatments once daily for ten consecutive days: CpdA (1 mg/kg); CpdA (3 mg/kg); dexamethasone (1 mg/kg) as gold standard; or the appropriate amount of water (negative control) (see Fig. 1a for experimental timeline). Safe dosage was determined and described in the aforementioned study (after reviewing other trials that included a systemic application of the drug) [15, 22, 23].

### Compound A preparation

Compound A, or 2-(4-acetoxyphenyl)-2-chloro-N-methyl-ethyl-ammonium chloride (Enzo Life Sciences, Lausen, Switzerland; chemical structure illustrated in Fig. 1b), was diluted in water for injection. Aliquots were frozen at −80 °C and thawed immediately before administration.

### Anesthesia

General anesthesia for auditory brainstem response (ABR) recordings and noise exposure was induced with medetomidine (0.3 mg/kg), midazolam (1 mg/kg), fentanyl (0.03 mg/kg), and ketamine (10 mg/kg). A half-dose booster injection was administered 1.5 h into noise exposure. Body temperature was maintained at 38 °C using a heating pad. Anesthesia was partially antagonized with atipamezole (1 mg/kg) towards the end of each procedure.

### Acoustic trauma

After five injections of the experimental treatment (to achieve sufficient drug level throughout both ears), guinea pigs were positioned on a rotating plate in a mac-2 soundproof chamber (Industrial Acoustics Company, Winchester, UK) and were exposed to 8–16 kHz octave-band noise at 115 dB for three hours. Sound was presented through a PH 8 Piezo tweeter horn (Conrad Electronic, Hirschau, Germany) positioned 5 cm from the animals’ pinnae and was amplified using an AMP75 wideband amplifier (custom-made by Thomas Wulf, Goethe University of Frankfurt, Frankfurt am Main, Germany). Noise calibration to target sound pressure level was performed before exposure sessions.

### Auditory brainstem responses

ABR thresholds were measured in the soundproof chamber described above, equipped with a DT-48 speaker (Beyerdynamic, Heilbronn, Germany) and a K2 microphone (Sennheiser, Wedemark-Wennebostel, Germany). The ear not receiving acoustic stimulation was plugged with Ohropax classic (Ohropax, Wehrheim, Germany) and a custom-made setup (Otoconsult, Frankfurt am Main, Germany) enabled auditory potential assessment [22]. Auditory stimuli included clicks and tone bursts (3 ms duration, 1 ms rise/fall, frequency 1–32 kHz, one step per octave). To obtain click thresholds, sound pressure was increased in 2 dB-steps, whereas 5 dB-steps were used for tone bursts (≤100 dB). Click- and tone burst-elicited signals were detected with a sample acquisition rate of 50 kHz, amplified (80 dB), band-pass filtered (10 Hz–10 kHz range), and averaged across 512 clicks and 256 tone bursts, respectively. Stimulus-evoked potentials were recorded a week prior to (pre-expo, baseline) and immediately after exposure (post-expo), as well as on days 1, 3, 7, 14, 21, and 28. Hearing thresholds were then independently analyzed by two investigators (L.D.L. & C.Z.) and were averaged between investigators and across ears. No response was rated as “maximum tested level +5 dB”.

### Histology

Animals were transcardially perfused with 4 % paraformaldehyde after audiometry on day 28. Cochleae were subsequently excised and fixed for at least 48 h and were randomly distributed across two groups for either organ of Corti whole-mount assessment or histological evaluation, respectively.

For histological evaluation, samples were first rinsed with distilled water and decalcified in 8 % ethylenediaminetetraacetic acid (Sigma-Aldrich, Vienna, Austria), and then embedded in paraffin for sectioning. Five 4 μm-thick sections were cut every 100 μm to the mid-modiolar plane; the rest of the cochlea was serially sectioned to include structures such as the round window membrane. Cochlear sections were then stained with hematoxylin-eosin and evaluated under a light microscope. A treatment-blinded histopathologist (H.S.) evaluated the tympanic membrane, wall of tympanic bulla and mucosal lining, round window membrane, and ossicles for exposure-induced changes. In addition, the spiral ligament, stria vascularis, and spiral ganglion neurons (SGNs) were assessed for nuclear hypercondensation of fibrocytes and pigmentation, intactness, and density in each of the 7 sections (across 3.5 half-turns) of Rosenthal’s canal (RC), respectively, in three mid-modiolar sections separated by 25 μm. Nucleated SGN profiles in each of the 7 sections of the RC (measured in mm^2^) were counted using Ellipse3D software (ViDiTo, Kosice, Slovakia). SGN density is reported as the average density across the three mid-modiolar sections. Due to the respective sectioning plane, the fourth middle and apical turns (see Wrzeszcz et al., for nomenclature) could not always be analyzed separately and were therefore excluded from the statistical analysis [24].

Organ of Corti whole-mounts were prepared by removing the otic capsule and then staining the tissue with Phalloidin-Tetramethylrhodamine B isothiocyanate (0.3 mg/ml PBS, Sigma-Aldrich, Vienna, Austria) and Hoechst 33342 trihydrochloride trihydrate (0.05 mg/ml PBS, Life Technologies, Carlsbad, CA, USA) for 30 min at room temperature. The cochlear turns were individually embedded in Fluorsave (Calbiochem, Darmstadt, Germany) and were observed by the blinded investigator (E.E.) under a confocal microscope to quantify the percentage of intact hair cells (HCs) in three randomly selected 200 μm-sections of each turn [25].

### Statistics

Data were analyzed using R 3.1.1 (R Foundation for Statistical Computing, Vienna, Austria) and are presented as mean values. Error bars reflect standard error of the mean. Two-way analyses of variance (ANOVAs) were performed, applying Tukey’s HSD correction for multiple comparisons. For histological data, contingency analyses were performed with the Freeman-Halton extension of the Fisher exact probability test [26]. *P*-values less than 0.05 were considered statistically significant.

## Results

### Compound A does not prevent threshold shift after noise exposure

**Fig. 2:**
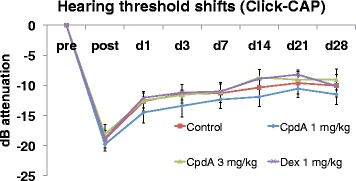
Click-ABR thresholds. Results of guinea pigs up to 4 weeks after noise exposure treated with the systemic application of water for injection (control, *square*), CpdA 1 mg/kg (*diamond*), CpdA 3 mg/kg (*triangle*), and dexamethasone 1 mg/kg (*x*). Error bars represent standard error of the mean. Abbreviations: pre = preexposure, post = postexposure, d = day, dB = decibels

Click threshold shifts were not significantly different across groups – thresholds ranged from −18.2 dB ± 5.3 dB (CpdA 3 mg/kg; AVG ± SD) to −19.8 dB ± 3.5 dB (CpdA 1 mg/kg) immediately after exposure to −9.0 dB ± 5.3 dB (CpdA 3 mg/kg) and −11.5 dB ± 5.4 dB (CpdA 1 mg/kg) at day 28 (see Fig. 2).

**Fig. 3:**
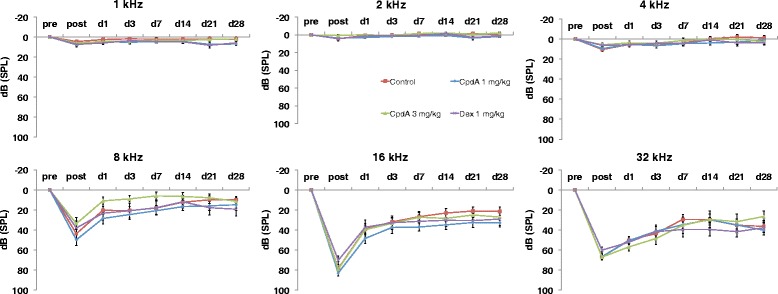
Pure-tone thresholds. Results of guinea pigs up to 4 weeks after noise exposure treated with the systemic application of water for injection (control, *square*), CpdA 1 mg/kg (*diamond*), CpdA 3 mg/kg (*triangle*), and dexamethasone 1 mg/kg (*x*). Error bars represent standard error of the mean. Abbreviations: pre = preexposure, post = postexposure, d = day, dB = decibels

Pure-tone threshold shifts were more prominent. As anticipated, noise exposure was immediately followed by threshold shifts specific to high frequencies (Fig. 3, 8-32 kHz, “post”), but thresholds at lower frequencies remained relatively stable (Fig. 3, 1-4 kHz, “post”, maximum shift of 10.4 dB ± 5.9 dB in the control group at 4 kHz). ANOVAs comparing thresholds between groups and across time revealed no significant differences in thresholds between animals that had received the negative control versus either of the CpdA dosages. Surprisingly, although the dexamethasone group did show better hearing thresholds than the other treatment groups at some frequencies immediately post-noise exposure (e.g., Fig 3, 16 kHz, “post”), the trend was not statistically significant. Spontaneous recovery (indicative of TTS) was observed in all groups to a certain extent, but damage persisted through day 28 (indicative of PTS).

### Noise exposure causes minimal hair cell loss in all groups

**Fig. 4:**
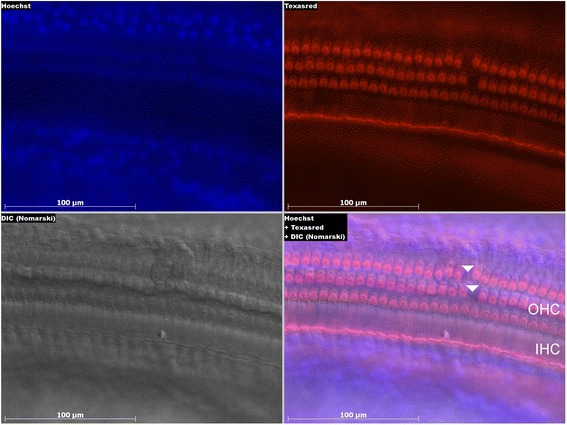
Confocal imaging in a control animal (water for injection) four weeks after noise exposure. Outer hair cell loss in the basal region marked with *arrowheads*. Abbreviations: Hoechst = Hoechst 33342 trihydrochloride trihydrate, DIC = Differential Interference Contrast, IHC/OHC = Inner/Outer Hair Cells

Outer and inner HCs analyzed from basal, second, and third turn and apical cochlear sections showed only slight HC loss without statistical significance across treatment groups (Table 1; Fig. 4), which supports the finding that hearing thresholds shifted minimally.

**Table 1:** Inner and outer hair cell counts (% ± standard deviation)

	Control	CpdA 1 mg/kg	CpdA 3 mg/kg	Dex 1 mg/kg
Inner Hair Cells				
Basal turn	100 ± 0.0	99.8 ± 0.4	99.8 ± 0.4	99.4 ± 0.9
Second turn	98.9 ± 2.5	100 ± 0.0	98.9 ± 1.7	99.2 ± 1.4
Third turn	99.7 ± 0.7	99.8 ± 0.4	99.7 ± 0.7	100 ± 0.0
Apex	100 ± 0.0	99.4 ± 1.0	100 ± 0.0	98.9 ± 1.5
Outer Hair Cells				
Basal turn	99.4 ± 0.8	99.4 ± 0.9	98.5 ± 1.8	98.9 ± 1.2
Second turn	98.2 ± 2.3	98.5 ± 2.0	98.5 ± 1.8	98.0 ± 3.2
Third turn	96.4 ± 4.3	93.5 ± 5.5	97.3 ± 2.2	95.6 ± 3.5
Apex	93.6 ± 5.3	94.8 ± 5.4	98.3 ± 2.1	94.0 ± 4.9

### Spiral ganglion neuron counts show no group differences

Spiral ganglion neuron integrity was also quantified (number of SGNs per mm^2^ per section of RC) but analyses revealed no statistically significant differences between the treatment groups (Control: *M* = 1250 ± 160; CpdA 1 mg/kg: *M* = 1360 ± 76; CpdA 3 mg/kg: *M* = 1250 ± 130; Dexamethasone: *M* = 1270 ± 90). Sub-analyses were performed, but did not reveal any frequency-specific between-group differences.

### Histological evaluation confirms ABR results

Middle ear assessment revealed small areas of tissue response to noise exposure (osteoneogenesis, fibrosis, and metaplasia of the bulla’s epithelial lining) in several animals, but these changes could not be linked to a specific treatment.

**Fig. 5:**
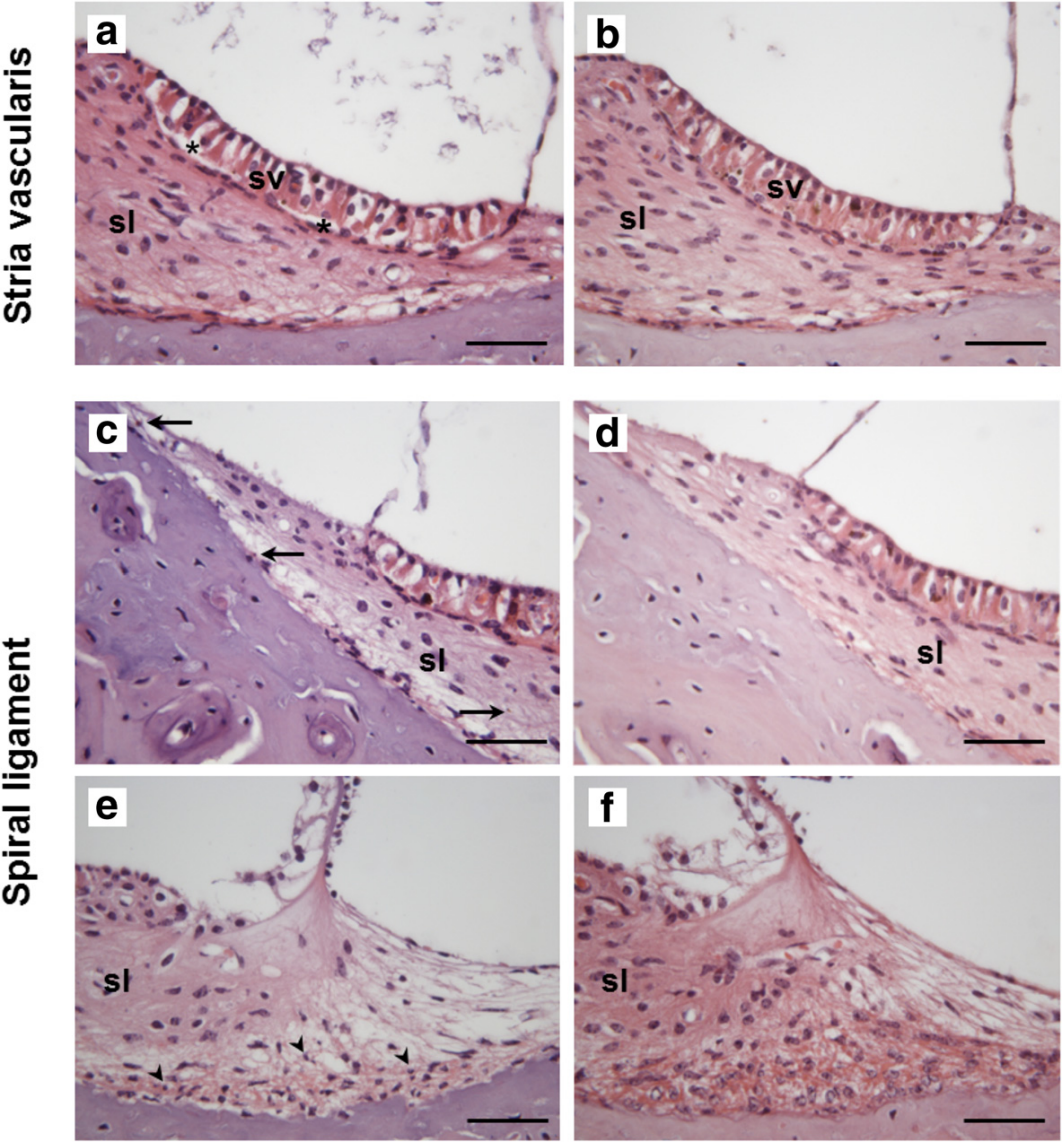
Representative sections of the cochleae of animals in different experimental groups. CpdA 3 mg/kg (**a**, **c**, **e**), control (**b**, **d**) or dexamethasone as the current gold standard (**f**). The histopathology of the stria vascularis (**a**, **b**) and spiral ligament (**c**-**f**) is depicted. **a** Detachment of the stria vascularis was noted in a high proportion of animals treated with CpdA. *Asterisks* (*) mark the gap between marginal/intermediate cells and basal cells of the stria vascularis (sv) bordering the spiral ligament (sl). **b** The stria vascularis of the majority of control and dexamethasone-treated animals did not show any signs of detachment. **c**, **d** Accumulation of pigment in the spiral ligament (arrows pointing to pigment granules) was seen in relatively more CpdA-treated animals (**c**), compared to control or dexamethasone-treated animals (**d**). **e**, **f** Nuclei of fibrocytes type III in the spiral ligament show a high degree of condensation (**e**, *arrow heads*), while appearing largely unaffected in control or dexamethasone-treated animals (**f**). Scale bars = 50 μm

Stria vascularis detachment, spiral ligament pigmentation, and fibrocytes type III showing nuclear hypercondensation were assessed in detail. Qualitative evaluation of the stria vascularis revealed intermediate and marginal cell detachment from the basal cell layer in 40 % of controls, 20 % of CpdA 1 mg/kg and 86 % of CpdA 3 mg/kg animals; however, none of the animals in the dexamethasone group showed detached stria vascularis (Table 2, Fig. 5, Fisher’s exact test: *p* = 0.02).

**Table 2:** Histological evaluation of inner ears (%, *p < 0.05)

	Control	CpdA 1 mg/kg	CpdA 3 mg/kg	Dex 1 mg/kg
Strial detachment 3^rd^ turn*	2/5 (40 %)	1/5 (20 %)	6/7 (86 %)	0/4 (0 %)
Pigmented spiral ligament*	3/5 (60 %)	2/5 (40 %)	6/7 (86 %)	0/4 (0 %)
Pyknotic fibrocytes type III				
Apical 2^nd^ turn and above Basal 2^nd^ turn and above	2/5 (40 %) 0/5 (0 %)	4/5 (80 %) 1/5 (20 %)	5/7 (71 %) 2/7 (29 %)	2/4 (50 %) 0/5 (0 %)

The dexamethasone group was also the only group to show no evidence of spiral ligament pigmentation (Control: 60 % contained pigment; CpdA 1 mg/kg: 40 %; CpdA 3 mg/kg: 86 %; Dexamethasone: 0 %) (Table 2, Fig. 5, Fisher’s exact test: *p* = 0.04).

Lastly, the number of fibrocytes type III showing nuclear hypercondensation varied between experimental groups. Few pyknotic nuclei of fibrocytes type III were detected in the second and third middle turns of control and dexamethasone-exposed cochleae (40 and 50 %, respectively); however, 80 % of CpdA 1 mg/kg animals and 71 % of CpdA 3 mg/kg exhibited condensed cell nuclei in these areas. Several also began to show signs of hypercondensation in the first middle turn (20 % in the 1 mg/kg and 29 % in the 3 mg/kg cohort) (Table 2, Fig. 5, Fisher’s exact test: *p* = 0.51 and 0.44, respectively).

## Discussion

Here we present the first evaluation of SEGRMs as an alternative to glucocorticoids for preserving hearing after noise trauma. Although not directly examining molecular pathways, the present experiments might provide insight regarding the mechanism of action of the latter class of drugs, which, despite their wide clinical application, remain poorly understood.

In our study, CpdA (a SEGRM) was compared with dexamethasone (positive control) and water (negative control) in its ability to preserve hearing after noise overexposure. Results indicate that neither dosage of CpdA (1 mg/kg vs. 3 mg/kg) provided physiological or anatomical protection that was significantly different from that offered by the negative control (water). However, CpdA and dexamethasone differed significantly in their abilities to preserve cochlear anatomy after noise exposure; specifically, while a detached stria vascularis was observed in CpdA-treated animals, the stria remained intact in dexamethasone-treated animals. It is well-known that acoustic overstimulation can trigger stria detachment and although the exact mechanisms driving this are unclear, studies investigating animal models of age-related hearing loss have proposed apoptotic cell accumulation in the stria’s basal layer as an explanation [27, 28]. Since strial degeneration plays an important role in age-related hearing loss in animals and humans, it is possible that long-term ABR threshold shifts would have been observed after our follow-up period of 4 weeks [29–32].

In addition to assessing the effects of treatment type on stria vascularis integrity, this study also investigated the state of fibrocytes in the different turns of the cochlea. These cells exist in many forms in the inner ear and were originally classified into four types according to location, orientation, immunostaining, and presence of transport-related enzymes (a fifth type was added several years later) [33, 34]. Fibrocytes type III are circumferentially located adjacent to the bone in the inferior region of the spiral ligament and, along with the spindle-shaped type IV fibrocytes (located lateral to the basilar membrane), serve to protect the cochlea against mechanical constraints induced by acoustic stimuli [33, 35]. Cell condensation and pyknotic nuclei are early signs of apoptosis and have been described in fibrocytes of the spiral ligament after noise overexposure in a mouse model [36]. Fibrocyte degeneration in the spiral ligament has been recognized as a major aspect of age-related cochlear degeneration. This pathology precedes HC and/or SGN loss, and potentially could have led to hearing loss after the end of our follow-up period [37, 38].

Contrary to what was expected, dexamethasone’s protective effect was minimal at best; in addition, HC loss across study groups was low. There are several possible explanations for these findings.

Wang et al. used a nearly identical experimental paradigm for their study analyzing the influence of dexamethasone on cochlear Hes1 expression [39]. After intraperitoneal drug injection for 5 consecutive days, guinea pigs were exposed to 8–16 kHz octave-band noise at 115 dB for three hours, which resulted in a threshold shift of approximately 30–35 dB (control) or 10–15 dB (dexamethasone) in the frequency range of 2–8 kHz after 24 h (higher frequencies were not assessed). They reported over 30 % of HCs missing in the basal turn in controls, while dexamethasone-treated animals showed 5 % HC loss in the same region. However, it has been suggested that pigmented guinea pigs (as used in our study) are less susceptible to noise than albino (used in the Wang et al. paper), possibly due to different distributions of glutathione S-transferase and glutathione peroxidase in the stria vascularis in these rodents [40–42]. Although other results (predominantly in mice) have led to controversial discussions about the extent of noise protection resulting from pigmentation, it has been established that pigment plays an important role in hearing not only in rodents but also in humans [43–45].

In light of this theory, the histological results are particularly interesting: the lack of spiral ligament pigmentation in the dexamethasone group could potentially have made these animals more susceptible to noise trauma.

Another factor contributing to the inconsistency between our results and those reported in the Wang et al. study is that the anesthetic dexmedetomidine (S-enantiomer of medetomidine - used in our study) has been found to be protective against noise-induced hearing loss [46, 47]. Overall, Wang et al.’s results were strong in comparison to findings from several other trials, which demonstrated only a moderate protective effect (i.e., slightly more pronounced than the present results) of glucocorticoids after noise exposure in guinea pigs [48, 49].

It is unclear whether using a more stable SEGRM than CpdA in a noise trauma model would lead to similar results, because CpdA is known to generate the alkylating pro-apoptotic metabolite N-methyl-2-(4-acetoxyphenyl)-aziridine in buffered solutions and – as our study group previously demonstrated – causes hearing loss when applied intratympanically [11, 22]. Based on the present histological data, which suggest increased damage in animals treated with the higher dosage of CpdA (Table 2), it is likely that other pharmacological candidates are more clinically promising regarding their abilities to exert otoprotective effects.

These experiments are the first tests of a novel class of drugs in the search for alternatives to glucocorticoids in otology. While the current trial’s results were negative (expected due to CpdA’s lability and narrow therapeutic range), the conclusions suggest an important role for glucocorticoid receptor dimerization and will hopefully help other researchers to expand the otological armamentarium [8, 11].

Since the GR’s ligand-binding domain is similar to that of the mineralocorticoid receptor (MR; 58 % identities, 76 % positives), both dexamethasone and CpdA can bind to it (albeit the latter with lower affinity) [50, 51]. Given the growing body of literature demonstrating major functions of the MR in the inner ear, the MR pathway may be relevant to the results of our study (suggesting that GR dimerization and/or MR activation might play a more important role than *transrepression* in biological processes in the inner ear) [52–54].

## Conclusion

While the SEGRM CpdA did not reveal substantial protective capacities when tested in a model of noise-induced hearing loss in guinea pigs, analysis of more candidates of this class of drugs – potentially with a more stable molecular structure – is warranted to determine whether *transactivation* (possibly together with MR effects) is more important than *transrepression* in the function of glucocorticoids in the inner ear. In addition to these mechanistic insights that will provide guidance for future directions of otoprotective drug discovery, such studies could represent the starting point for the eventual clinical application of glucocorticoid analogs with a more favorable side effect profile for inner ear therapy.

## Abbreviations

ANOVAs: analyses of variance
CpdA: compound A
GR: glucocorticoid receptor
GRE: glucocorticoid response element
HC: hair cell
MR: mineralocorticoid receptor
PTS: permanent threshold shift
RC: Rosenthal’s canal
SEGRM: selective glucocorticoid receptor modulator
SGN: spiral ganglion neuron
TTS: temporary threshold shift
